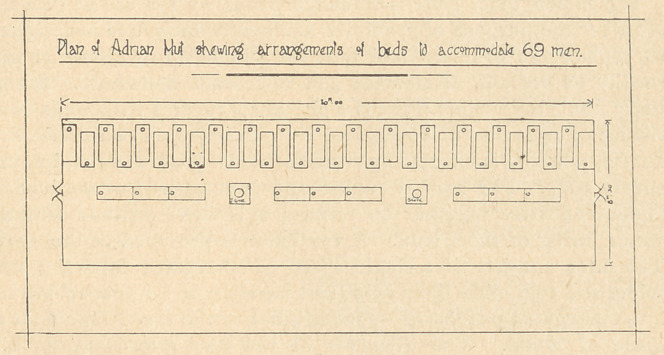# Considerations in the Prevention of Respiratory Affections

**Published:** 1918-10

**Authors:** W. W. O. Beveridge


### ACUTE RESPIRATORY INFECTIONS

**Considerations in the Prevention of Respiratory Affections.** By Colonel W. W. O. Beveridge, C. B. D. S. O.

The speaker said in part :

The spread of infectious diseases affecting the respiratory tract is influenced in army life by a variety of circumstances of which overcrowding is probably one of the most important.

Overcrowding has always been recognized as a prominent factor in the spread of disease both in civil and military life. In England as long ago as 1861 the Royal Commission for Improving the Sanitary Condition of Barracks commented as follows :

" Before the soldier can be assured of having the amount of space required for health, there must be a distinct recognition that the amount given by regulation (i. e. 600 cubic feet) is on no account to be tampered with. No increase of regimental strength, no want of store rooms, libraries, or reading rooms, should for an instant be permitted to interfere with it.

" It would never be pleaded, as a reason for reducing the soldier's ration of bread and meat, that a larger number of men had oined the regiment than the commissariat could provide for.

" Why should the soldier's air ration, which is [equally important for his health and efficiency, be differently dealt with?

" In any case overcrowding should utterly be put an end to.

" They have not been aware that, if above a certain number of men are placed in a given cubic space, the lives of some of these men, and the health of others, is certain to be sacrificed. "

Accordingly during the period prior to the war accommodation in barracks and hutments in Great Britain was provided on the scale of 60 square feet and 600 cubic feet per man. On the outbreak of the present war the great influx of recruits necessitated a somewhat less generous provision, and having regard to military exigencies 40 square feet or 400 cubic feet was fixed as the minimum accommodation which could safely be permitted in quarters. This has since come to be regarded as a war time standard which every effort should be made to attain.

All epidemiological experience points to the very marked effect which overcrowding exerts upon the spread of infectious diseases, especially those affecting the respiratory tract. It is a matter of common knowledge that when diphtheria occurs among men who are crowded together, " carriers " of Klebs-Loeffler bacilli are usually numerous, and when these men are spread out in well ventilated quarters the " carrier " condition rapidly disappears.

The evil effects of crowding together in ill-ventilated wards, patients suffering from measles, are well known; the severity of the disease increases, and deaths from pneumonia as a complication may reach a high figure. Should such a state of affairs occur, experience has shown that it can be immediately remedied by the simple procedure of providing sufficient space and adequate ventilation.

Similary outbreaks of cerebro-spinal fever depend largely upon overcrowding and ill ventilation. Many such outbreaks have been checked by remedying these conditions. At a meeting of the permanent section of the Allied Sanitary Conference in June, 1917, Professor Rho (Italy) stated that during a volcanic eruption 30,000 men were placed under canvas for a long period during cold and inclement weather They were not overcrowded and enjoyed complete freedom from cerebro-spinal fever, although an outbreak of this disease was raging in barracks in the vicinity which were manifestly overcrowded. In this connection an interesting and important investigation into the relationship which exists between overcrowding and the meningococcus carrier rate has recently been carried out by Captain J. A. Glover, R. A. M. C., in England. The conclusions which he arrives at are definite and quantitative and afford a useful confirmation of the conclusions which have been arrived at in the past by epidemiologists and others as a result of experience and observation. Captain Glover points out that a high carrier rate of virulent meningococcus strains is probably one of the chief factors tending to produce outbreaks of cerebro-spinal fever so that a high incidence of carriers may reasonably be taken as an index to the likelihood of an outbreak occurring, other conditions being favorable.

By the simple expedient of allowing a space of 2 1/2 feet between each bed and by improving the ventilation of the quarters, the carrier rate, in one instance, fell from 28 to 2 per cent in 9 weeks, from 28 to 7 per cent in 6 weeks in another, from 38.5 per cent to 4.5 per cent in 6 weeks in a third, and from 28 per cent to 4.5 per cent in 5 weeks in a fourth.

A large number of similar experiments were carried out, all of which support the view that the distance between beds is of paramount importance and that quite a moderate degree of " spacing out " of beds combined with simple methods for improving ventilation is highly effective in reducing carrier rates.

Infectious diseases affecting the respiratory passages are largely spread by " spraying " from the nasal and buccal orifices of infected persons, so that the chances of a healthy person in the neighborhood becoming infected increases enormously the nearer such a person is to an infected individual. In addition, inadequate ventilation permits of a greater concentration of infective material in the surrounding air which increases the chances of transmission of infection. The important elements in " overcrowding ", so far as the spread of infectious diseases affecting the respiratory passages is concerned, are, therefore, proximity of heads and defective ventilation. Ventilation may be improved within the limits short of creating a draught, but satisfactory results can only be expected when close proximity of heads is also prevented.

Proximity of heads and degree of ventilation are, therefore, the criteria by which the adequacy of accommodation should be judged.

Floor space may not give trustworthy information as to the actual available accommodation; the presence of doors or awkward angles in rooms may lead to overcrowding, although the total floor space, if it were all available, would be adequate.

In calculating cubic space in quarters, heights above 10 feet are neglected as not contributing useful space so far as the prevention of true overcrowding is concerned. In hutments the cubic space above 10 feet from the floor is usually relatively small and for practical purposes may be neglected. On this basis the floor space expressed in square feet is numerically 1/10th of the cubic space, so that accommodation may, for simplicity, be expressed in terms of square feet of floor space. Where huts are about 20 feet wide, as is commonly the case, and men sleep down each side the linear wall, space per man is numerically a tenth of the floor space per man and affords a ready and rapid guide as to the sufficiency of accommodation. In ordinary hutments, therefore, 400 cubic feet or 40 square feet of floor space or 4 linear feet of wall space per man may be regarded as affording the same accommodation.

Quite apart from convenience of measurement, linear wall space gives information in regard to the most important factor in accommodation, namely proximity of heads. It sometimes happens, for instance, in circular huts and shelters that it is impossible to give what is usually considered an adequate amount of floor space but that there is no difficulty in providing a suitable amount of wall space, thus ensuring a satisfactory distance between heads. In addition to adequate spacing between heads, it is of course desirable to obtain as much floor space and cubic space as possible, but where owing to military exigencies this cannot be done every endeavor should be made to obtain a sufficient amount of the most essential factor in the prevention of overcrowding, namely wall space.

Accommodation in tiers is not recommended where it can be avoided; it may be necessary in forward areas but in no case should more than two tiers be countenanced unless for very short periods and on grounds of military urgency.

Hutting accommodation for the British Army on the Lines of Communication in France is provided on a basis of 4 feet of wall space per man, unless the huts are more than 20 feet wide, when 40 square feet of floor space per man is allowed. This includes accommodation for Labor Companies, both white and colored, and for prisoners of war and their escorts. Vigilance is exercised in preventing more men than the authorized number from being accommodated in any hut, particularly in the case of colored laborers who are specially susceptible to respiratory diseases.

In ordinary hospitals 60 square feet of floor space with six feet of wall space per patient is provided, but during periods of pressure this may have to be reduced to five feet of wall space. In hospitals for infectious disease 100 square feet of floor space is allowed.

Several types of huts for the accommodation of troops have been erected, the majority of which vary from 16 to 20 feet in width.

The Adrian Hut is constructed in sections of 2 meters length which can be bolted together. Its extreme width at floor level is 27 feet 4 1/2 inches. A hut 30 metres long can accommodate 69 men giving each approximately 40 square feet of floor space, if the precautions referred to below are observed. Thirty beds could be placed down each side, and owing to the great width of the hut 9 can be arranged end to end down the centre. In order to make the best use of the available floor space it would be necessary for alternate men in the beds along the wall to sleep with their heads projecting towards the centre of the room. This arrangement could still be further improved by drawing alternate beds one foot away from the wall (see figure). To ensure this arrangement being adhered to, it would be necessary to fix the beds to the floor and to make the head end of each bed slightly higher than the foot end so that men would be constrained to sleep with their heads at the proper ends. The difficulties of carrying out these refinements in actual practice are obvious. It has also been suggested that risk of spread of infection might be diminished by the erection of small partitions or screens between each bed.

In army areas variations in the patterns of hut have lately been restricted for the sake of simplicity and convenience; the following are typical examples of huts utilized in army areas as shelter for troops.

| | | |
|---|---|---|
| Adrian Huts . . . | 30 meters. . . . . | Men or Institutes. |
| Standard Hut . . | 60′ × 16′ . . . . . | Men or Institutes. |
| Nissen Bow. . . . | 26′10″ × 15′8″. . . | Men. |
| Nissen Steel Tent. (Circular). . . . | Diameter 14′6″ 1/2. | Men. |

The Nissen Steel Tent is intended primarily for use in forward areas for the purpose of providing shelter for men, and is designed to replace the ordinary bell tent for winter occupation. It can readily be erected, dismantled and re-erected elsewhere. Essentially the Nissen Steel Tent is a circular structure with walls consisting of 20 sheets of corrugated iron arranged on end vertically. Lateral junction is effected by means of metal grooves in which the sheets slide. The roof is conical and is composed of sections of sheet iron fitted together on rafters; it is also furnished with an inner lining of sheet iron. A ventilator is provided at the apex. The door consists of a single sheet of corrugated iron 2′ 3 1/2″ wide, and two oiled linen windows which can be opened are let into corrugated panels on opposite sides of the tent. The floor is made of wood. The diameter of the steel tent is 14′ 6 1/2″, about 2 feet more than an ordinary bell tent. The tent accommodates 11 men, allowing 4 feet of wall space to each, which is greater than that usually given to men in ordinary bell tents. The cubic capacity of the steel tent is also considerably greater than that of a bell tent, owing to its high vertical sides.

Ordinary circular bell tents, diameter 12′ 6″, usually accommodate not more than 12 men, but on occasion 14 men may be placed in a tent. The wall space, floor space, and cubic space are thus below the standard prescribed for hutted accommodation. In warm weather when the tent flies are kept open, this is partly compensated for by free ventilation, but during wet and cold weather when the tent is closed the conditions both in regard to proximity of heads and ventilation are such as would favor the spread of disease affecting the respiratory passages. Owing to the men being grouped in separate tents, the danger of spread of disease is limited and the risk of an extensive outbreak is not so great as if the same degree of overcrowding occurred in a large hut or barrack room.

It is obvious from what has already been said that overcrowding is a relative matter and no definite line of demarcation can be drawn between ample space and overcrowding; the two merge gradually into one another. For practical purposes, however, a compromise between military necessity and the ideal has to be made. The minimum standard of accommodation already referred to represents the practical compromise which has been effected; less accommodation than this increases enormously the chances of spread of infectious disease affecting the respiratory tract.

The speaker alluded briefly to some other factors which have a bearing on the spread of respiratory diseases.

The procedure adopted in dealing with contacts and carriers varies somewhat according to circumstances and to the disease in question. Cerebro-spinal fever and diphtheria may be regarded as infectious respiratory diseases and the mode of dealing with contacts and carriers of these diseases may be briefly considered.

Contacts with a case of cerebro-spinal fever are submitted to a bacteriological examination with a view to discovering carriers. All contacts who give negative results are released; those who give positive results are detained and suitably treated with antiseptic douches or sprays until two successive bacteriological examinations at several days' interval are negative.

Contacts with a case of diphtheria are examined bacteriologically and all carriers isolated in roomy well-ventilated quarters until bacteriological examination gives negative results. In addition local treatment of throat and naso-pharynx may be required. Specific prophylactic measures, such as the administration of antidiphtheritic serum to contacts, are not usually undertaken. Very few contacts or carriers if properly isolated and treated develop diphtheria, and, since they are kept under observation, any who might develop clinical symptoms would immediately receive a curative dose of anti-diphtheritic serum. Schick's reaction makes it possible to detect those in whom natural immunity is low and a degree of active immunity can be induced by the administration of neutralized diphtheritic toxin. This procedure is, however, hardly adapted for general use in the field, and in view of the ease with which outbreaks of diphtheria can be controlled by the simple precautions already described, specific prophylactic measures have not been found necessary as a routine procedure.

In conclusion it may not be out of place to refer to certain general considerations which are important in so far as they aid in maintaining resistance to infection. Protection against cold and inclement weather in the shape of warm and waterproof clothing has a marked effect in preventing a lowering of resistance, and in this connection the importance of providing efficient drying rooms at all camps and billeting areas cannot be overemphasized. Such drying rooms may be heated by coke or coal stoves, or the heat from incinerators may be utilized.

In order to maintain resistance to disease and to mitigate its severity when contracted, a sufficiency of good and well-cooked food is essential. The British Field Service Ration provides a daily energy value of rather more than 4,000 calories and contains 166 grams of fat, a highly important food element. There is no doubt that this adequate diet has a material influence in maintaining resistance to disease and is in part responsible for the low incidence of respiratory diseases throughout the British Armies in France.

## Figures and Tables

**Figure f1:**